# Folk prescription for treating rhinitis as a rare cause of childhood lead poisoning: a case series

**DOI:** 10.1186/s12887-018-1193-9

**Published:** 2018-07-06

**Authors:** Xiao-Lan Ying, Morri Markowitz, Chong-Huai Yan

**Affiliations:** 1 MOE-Shanghai Key Laboratory of Children’s Environmental Health, Xinhua Hospital affiliated to Shanghai Jiaotong University School of Medicine, Shanghai, 200092 China; 2 Division of Environmental Sciences, Children’s Hospital at Montefiore, Albert Einstein College of Medicine, Bronx, NY10467 USA

**Keywords:** Child, Lead poisoning, Folk medicine, Nasal spray, Rhinitis

## Abstract

**Background:**

Folk prescriptions continue to be important sources of childhood lead poisoning. Nasal spray folk prescriptions for treating rhinitis has only been reported once previously as a cause of lead poisoning.

**Case presentation:**

We identified three pediatric cases of severe lead poisoning caused by nasal spray folk medicines prescribed for treating rhinitis. The three patients had similar clinical manifestations including: severe abdominal pain, headache, pale appearance and fatigue. Liver function tests were abnormal. Blood lead levels (BLLs) of the three patients were 91 μg/dL, 91 μg/dL, and 105 μg/dL, respectively. After chelation BLLs decreased. The lead content of the three folk remedies as measured by inductively coupled plasma mass spectrometry (ICP-MS) were 14.8, 22.3, and 33.4%. All the symptoms resolved during a course of chelation therapy. There were no severe side effects of treatment.

**Conclusions:**

Nasal spray folk prescriptions for treating rhinitis may contain extremely high bio-accessible lead content and are potential sources of lead poisoning. Clinicians should be alert to this possibility especially in those children presenting with multisystem symptoms.

## Background

Eliminating pediatric lead poisoning remains a worldwide challenge [1, 2]. Since symptoms of lead poisoning are nonspecific, such as abdominal pain, pale appearance, behavioral changes, headaches, fatigue, and irritability [3], the possibility of lead poisoning is easily overlooked. Thus the correct diagnosis can be delayed resulting in progression of toxicity.

The accurate identification of at-risk populations from unusual sources of lead exposure is essential to prevent further toxicity. Some cases of lead poisoning caused by folk medicines have been reported in recent years [4–6]. However, to our knowledge, only one case of lead poisoning due to a nasal spray folk remedy has been reported [7].

Extremely elevated BLLs (BLLs ≥70 μg/dL) now occur rarely in children [8]. We report three cases of severe childhood lead poisoning from folk medications specifically prescribed for treating rhinitis. These cases were complicated by concurrent abnormal liver function which can affect lead poisoning treatment.

## Case presentation

### Case 1

A 7-year-old boy was referred to our hospital for an elevated BLL (> 60 μg/dL) discovered during routine screening procedures. On admission, a recheck of the BLL, tested by Atomic Absorption Spectrometry (AAS), showed the level to be 91 μg/dL. Thus the diagnosis of lead poisoning was confirmed.

Two months before admission, he started to feel dizzy and developed headaches. Symptoms progressed to poor appetite, mouth-bitterness, repeated vomiting and abdominal pain for more than a month. The abdominal pain was intermittent, without an obvious precipitant and generally lasted for 10 min with spontaneous resolution. Subsequently, intense joint pain and fatigue occurred causing him to be unable to walk by himself.

Before admission, he had been hospitalized twice elsewhere. At his first presentation 2 months earlier, laboratory examinations found elevated serum liver enzymes: alanine transaminase (ALT) 145 U/L and aspartate aminotransferase (AST) 78 U/L. He also had anemia with a hemoglobin (Hb) level of 96 g/L and red blood cell (RBC) count of 3.67 × 10^12^/L). Superficial gastritis and bile reflux were found by endoscopy. An upper abdominal CT angiography showed “a general decrease in liver density; possible superior mesenteric artery syndrome”. A descriptive diagnosis of “chronic superficial gastritis, possible superior mesenteric artery syndrome, and abnormal liver function tests” was made. He was treated with omeprazole and sucralfate for 2 weeks which was accompanied by relief of his symptoms. He was discharged from the hospital without an identified etiology.

Ten days after discharge, he was admitted to another hospital for intermittent vomiting and severe abdominal pain. Liver function tests, electroencephalogram and abdominal ultrasonography were normal. An incidental BLL test was performed (this hospital tests lead routinely) and reported as elevated. He was referred to our hospital for further evaluation and treatment.

**Fig. 1:**
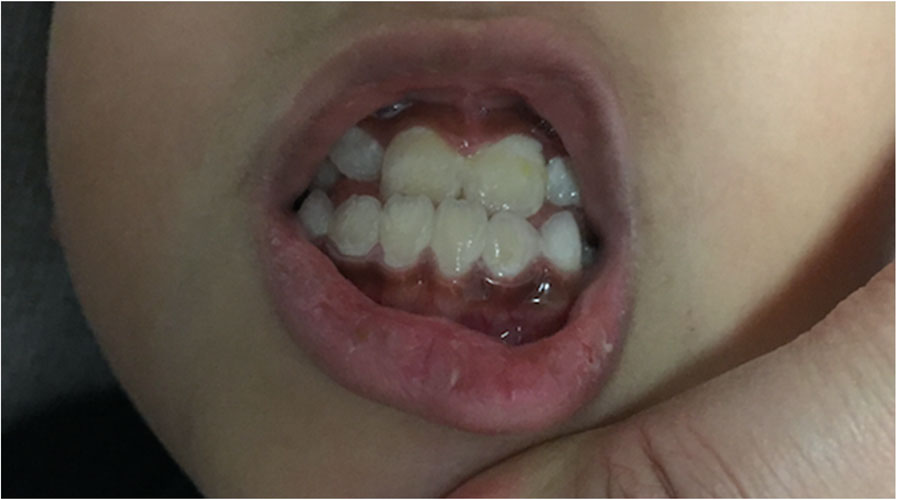
Gum lead line

**Fig. 2:**
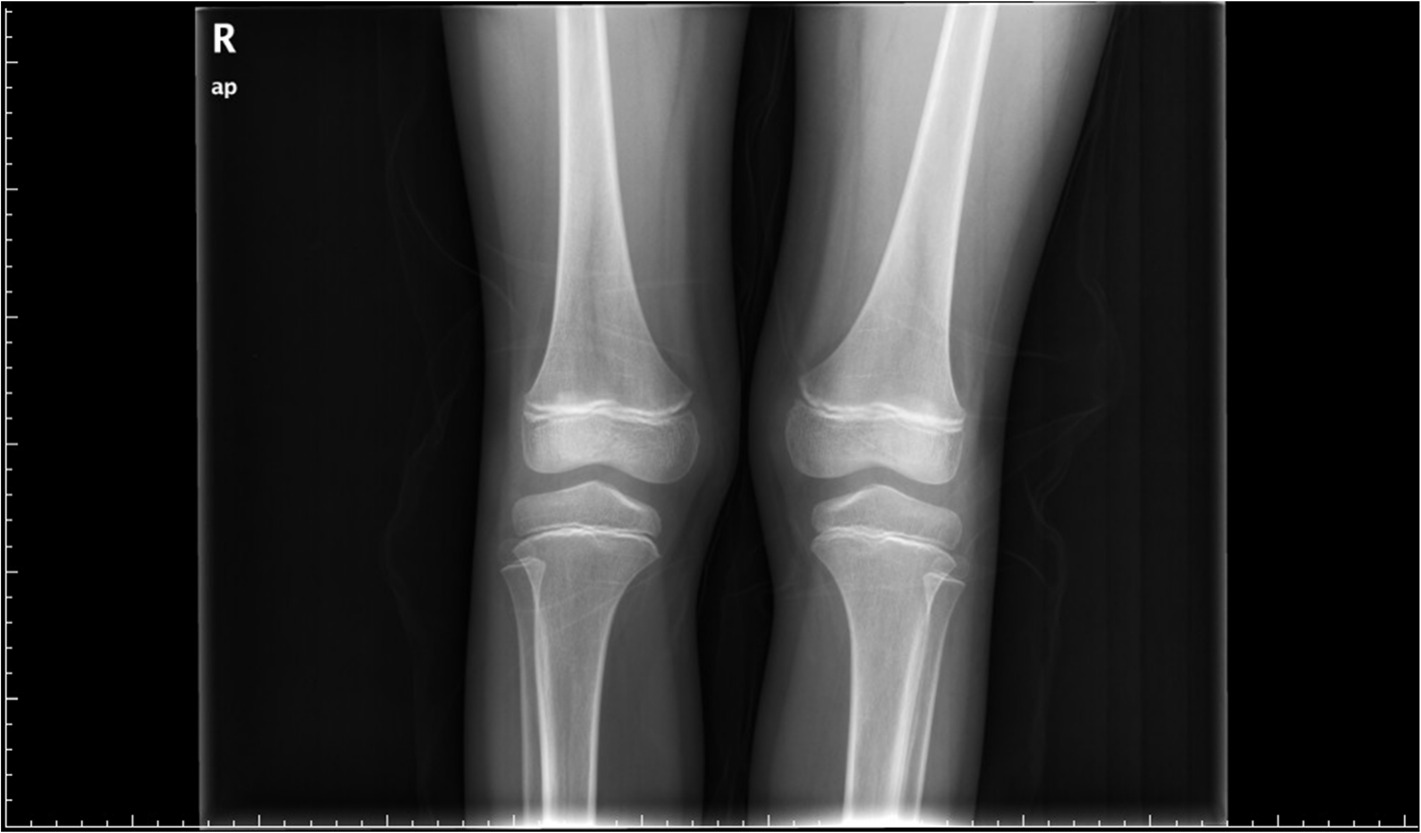
Anteroposterior radiograph of the knees bilaterally

On admission to our hospital, his examination found a soft, non-tender abdomen without rebound tenderness. Both liver and spleen were impalpable. Neurologic examination was normal. Abnormally increased pigmentation of the gums (“lead line”) was observed (Fig. 1). No radiopaque point masses were identified on anteroposterior abdominal radiography. Anteroposterior radiograph of the knees (Fig. 2) showed increased linear radio-density at the distal femoral and proximal tibial and fibular metaphyses. Serum chemistries were normal. The RBC count was 3.68 × 10^12^/L (reference range 4.0–5.5 × 10^12^/L) and the Hb level was 101 g/L (reference range 120-150 g/L), consistent with anemia.

**Fig. 3:**
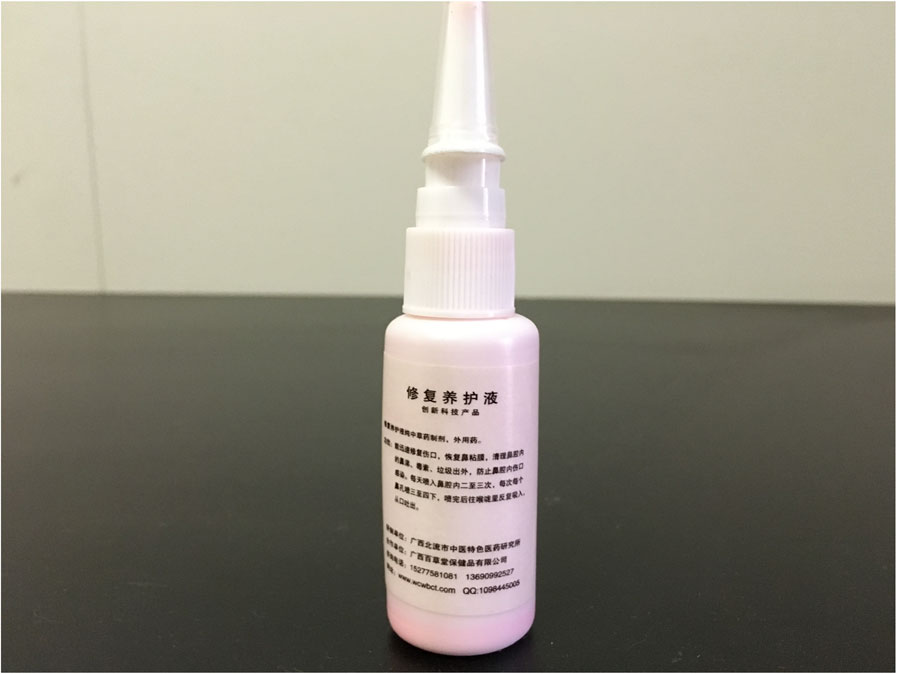
Folk medicine for treating rhinitis

A more detailed history about sources of lead exposure was taken. He had been using a red-colored folk medicine (Fig. 3), spraying it in the nostrils two to four times a day during the week prior to consulting a doctor. The nasal spray liquid was obtained from a traditional Chinese medicine (TCM) practitioner (Shenzhen, Guangdong, China). Analysis of the liquid revealed a lead concentration of 148,000 mg/kg (14.8%) as measured by ICP-MS.

Chelation therapy combined orally administered dimercaptosuccinic acid (DMSA) (350 mg/m^2^ body surface area, q8h) and intravenous calcium disodium edetate (CaNa_2_EDTA) (1 g/m^2^ body surface area, continuous infusion using a micro-pump over 24 h) for 5 days. Glutathione (600 mg, intravenous drip, once a day) was added as an antioxidant. All the symptoms resolved; and the BLL declined to 21 μg/dL after chelation therapy was completed.

### Case 2

**Fig. 4:**
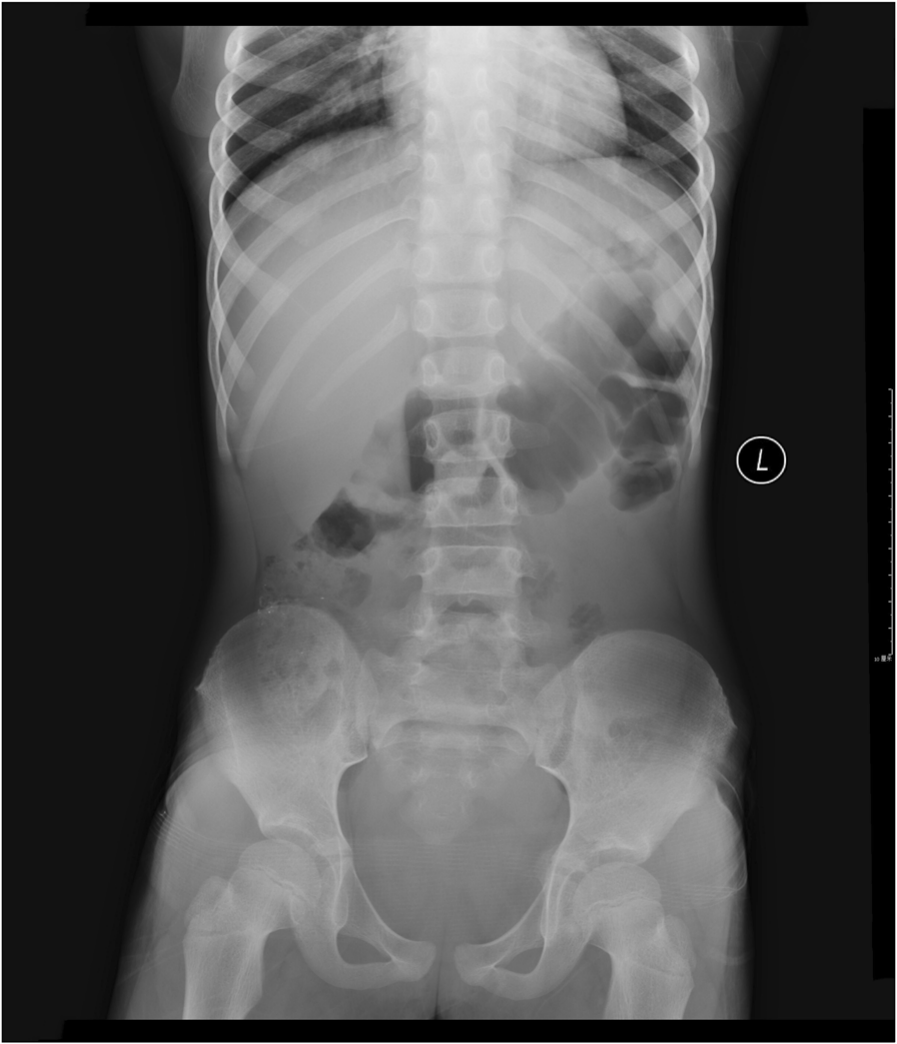
Radiography of the abdomen

An 8-year-old girl with rhinitis had consulted the same TCM practitioner as in case 1 and was prescribed the same nasal spray liquid to be used twice a day for 10 days. She developed severe abdominal colic, vomiting, constipation and felt fatigued. Her venous BLL was 91 μg/dL. The nasal spray contained 223,000 mg/kg (22.3%) lead. Abnormal laboratory test results included: creatine kinase 747 U/L (reference range 30–135 U/L), creatine kinase isoenzyme MB 14.8 ng/mL (reference range 0–6.8 ng/mL); AST 119 U/L (reference range 8–38 U/L), ALT 390 U/L (reference range 0-75 U/L). Radiography of the abdomen revealed shadow of stool and gas as well as points of increased density (Fig. 4).

It could not be determined whether the radiopaque particles seen on the abdominal x-ray contained lead or not. Since chelating agents may increase gut lead absorption, folium sennae was administered as a cathartic to eliminate lead from the intestine prior to initiating chelation. The chelation regimen was identical with case 1. However, after 2 days of therapy the white blood cell count fell to 2.11× 10^9^/L (normal range: 4.0–10.0× 10^9^/L). This may be attributed to DMSA, which was withheld subsequently. Chelation continued with an intravenous infusion of CaNa_2_EDTA to achieve a BLL of 36 μg/dL at the end of 5 days.

### Case 3

A 5-year-old boy had consulted the same TCM practitioner as in Cases 1 and 2 and was prescribed the same medication to be used twice a day for 7 days. Severe abdominal pain and vomiting followed. Prior to transfer to our hospital his initial laboratory data showed markedly elevated liver enzyme levels (ALT 1083 U/L, AST 972 U/L). No abnormality was found on abdominal x-ray. On examination, his liver was palpable 4 cm below the right costal margin. The BLL on admission was 105 μg/dL. The nasal spray contained 33.4% lead.

Chelation therapy was initially withheld because of the severely altered liver enzyme results as both drugs are potentially hepatotoxic. Initially, he received treatment aimed at improving liver function with glutathione and disodium glycyrrhetate (Table 1) and continued to receive these medications during the whole course of treatment. As his liver function tests improved, his BLL went down concomitantly, prior to chelation (Table 1, BLL of Day 1: 105 μg /dL; Day 4: 96 μg/dL; Day 8: 80 μg/dL) at which point chelation treatment was initiated with DMSA and CaNa_2_EDTA infusion (same usage as Case 1). The post-chelation lead level for this child was 34 μg/dL. No adverse events happened to all the three children.

**Table 1:** Liver function test during therapy

Variable	Reference range	Day1^a^	Day 2	Day 3	Day 4	Day 5	Day 6	Day 7	Day8^b^	Day9	Day10	Day13
ALT (U/L)	0–75	641	521	415	317	254	197	176	117	124	14	67
AST (U/L)	8–38	280	137	102	91	85	75	81	57	93	23	40

^a^Day 1: Start to get liver-protecting treatment in our hospital

^b^Day 8: Start chelation therapy combined with liver-protecting treatment

## Discussion

### Absorption of lead

Ingestion and inhalation are the two primary modes of lead entry into the body. The former is more common in children due to hand-to-mouth activity, while the latter occurs more frequently in occupationally exposed adults [9]. The percentage of lead absorbed from the gut depends on factors as follows: particle size, gastric pH, other material in the gut, and nutritional status of essential elements [10]. Inhalational absorption through the lung depends on particle size.

The three patients had reportedly short-term exposures to nasal spray liquids for treating rhinitis that had extremely high lead concentrations. The short period of exposure is consistent with acute lead poisoning. This folk remedy was sprayed directly into the nostrils. All three children mentioned that they tasted and swallowed the liquid. Thus, the lead was potentially absorbed from multiple sites including the nasal mucosa, the lungs, as well as the gastrointestinal tract.

### Diagnosis of lead poisoning

Abdominal pain caused by lead poisoning has been reported [11, 12]. Possibly the delay in diagnosis in our patients was due to a lack of awareness by the physicians of its features in children. This highlights the need to educate clinicians including subspecialists about this disease. Lead poisoning should be in the list of possible diagnoses for children with unexplained multi-systems symptoms, especially of the gastrointestinal and central nervous systems.

These children also had detectable signs of lead poisoning. The line of gingival hyperpigmentation [13] in the first patient has been described in occupationally lead exposed adults [14]. He also had a “lead line” on long bone radiography. The width of the dense metaphysis has been reported to correlate with the length of the period of toxic exposure [15].

### Folk remedy as a potential source of lead poisoning

Lead poisoning can be diagnosed through the determination of the lead concentration in whole blood. In children, an elevated BLL indicates the need to identify the sources of exposure that likely lead to ingestion. While typical environmental sources predominate as the cause of lead poisoning, cultural factors are also important. Besides lead-containing paint, various sources of lead were identified for children with BLL ≥ 45 μg/dL in New York City: imported spices, cosmetics, as well as traditional folk medicines [8]. A detailed history about the usage of traditional folk medicine is essential. Laboratory analyses of these medicines will then determine the lead content.

Lead poisoning due to folk remedies for treating skin problems [16], abdominal pain [17], epilepsy [18], or “strengthening eyes” [19] have been reported before. However, a nasal spray folk remedy for treating rhinitis as a potential source of lead poisoning has only been reported in a single adult [7]. As shown in Fig. 3 the container can look completely ordinary for a nasal spray and is clearly not an indicator of safety. Usage of such products in children should prompt clinicians to consider lead screening and public health agencies to educate local communities of the risks of their use.

Historically, the compositions of folk medicines were proprietary and medicine containers did not indicate the constituents. Given the high concentrations that we found it’s likely that the Traditional Chinese Healer purposefully added lead to the nasal spray, presumably because he felt it offered a therapeutic benefit. Given the known and potentially lethal toxicity of lead, its medicinal use should be prohibited. These cases highlight the need for governmental oversight of folk medicine content.

### Treatment of lead intoxication

Despite intestinal and neurological symptoms the diagnosis of lead poisoning was delayed. Hepatotoxicity was observed in our patients as evidenced by elevated transaminase levels. Both DMSA and CaNa_2_EDTA can be hepatotoxic; pretreatment evaluation of liver function is necessary. With DMSA, a transient mild rise of hepatic transaminases is the most common adverse effect reported, generally resolving on discontinuation of treatment without long-term sequelae. Published studies on the frequency of this transaminase increase differ, varying between < 1.5 and 60% of patients [20, 21]. In a multicenter placebo controlled DMSA trial conducted in children with initial BLLs between 20 and 45 μg/dL, no difference in the rate of elevated ALT levels in treated versus untreated children was observed [22].

Although hepatic damage is not a common feature of childhood lead poisoning [23, 24], animal studies have found that chronic lead poisoning caused mild histological and histochemical changes in the liver [25]. The very high liver enzyme levels in case 3 delayed chelation. With the conservative approach taken, liver function recovered quickly and BLLs declined. Spontaneous excretion of lead in patients with acute lead poisoning may have a greater impact on BLLs than those with more chronic lead poisoning since lead has not had time to accumulate in bone.

## Conclusions

Although the differential diagnosis of lead poisoning is broad, any child who presents with unexplained GI and CNS symptoms should undergo a BLL test, which will directly support or reject the diagnosis of lead toxicity.

Folk remedies for treating rhinitis can be an unexpected source of lead exposure. It is incumbent on pediatricians to perform a diligent search for unconventional sources of lead when confronted with cases without an apparent etiology.

## Abbreviations

AAS: Atomic absorption spectroscopy
ALT: Alanine transaminase
AST: Aspartate Aminotransferas
BLLs: Blood lead levels
CaNa_2_EDTA: Calcium disodium edetate
DMSA: Dimercaptosuccinic acid
Hb: Hemoglobin
ICP-MS: Inductively coupled plasma mass spectrometry
RBC: Red blood cells
TCM: Traditional Chinese medicine
